# Prevalence and characteristics of coronary artery anomalies in an adult population undergoing multidetector-row computed tomography for the evaluation of coronary artery disease

**DOI:** 10.1186/s12872-015-0098-x

**Published:** 2015-10-02

**Authors:** Christos Graidis, Dimokritos Dimitriadis, Vasileios Karasavvidis, Georgios Dimitriadis, Efstathia Argyropoulou, Fotios Economou, Dadoush George, Antonios Antoniou, Georgios Karakostas

**Affiliations:** Department of Interventional Cardiology, Kyanous Stavros Hospital, Vizyis-Vyzantos 1 Street, Thessaloniki, 54636 Greece

**Keywords:** Coronary artery anomalies, Coronary angiography, Multi-detector computed tomography

## Abstract

**Background:**

Congenital coronary anomalies are uncommon with an incidence ranging from 0.17 % in autopsy cases to 1.2 % in angiographically evaluated cases. The recent development of ECG–gated multi–detector row computed tomography (MDCT) coronary angiography allows accurate and noninvasive depiction of coronary artery anomalies.

**Methods:**

This retrospective study included 2572 patients who underwent coronary 64-slice MDCT coronary angiography from January 2008 to March 2012. Coronary angiographic scans were obtained with injection of 80 ml nonionic contrast medium. Retrospective gating technique was used to synchronize data reconstruction with the ECG signal. Maximum intensity projection, multi-planar reformatted, and volume rendering images were derived from axial scans.

**Results:**

Of the 2572 patients, sixty (2.33 %) were diagnosed with coronary artery anomalies (CAAs), with a mean age of 53.6 ± 11.8 years (range 29–80 years). High take-off of the RCA was seen in 16 patients (0.62 %), of the left main coronary artery (LMCA) in 2 patients (0.08 %) and both of them in 2 patients (0.08 %). Separate origin of the left anterior descending artery (LAD) and left circumflex artery (LCx) from left sinus of Valsalva (LSV) was found in 15 patients (an incidence of 0.58 %). In 9 patients (0.35 %) the right coronary artery (RCA) arose from the opposite sinus of Valsalva with a separate ostium. In 6 patients (0.23 %) an abnormal origin of LCX from the right sinus of Valsalva (RSV) was found with a further posterior course within the atrioventricular groove. A single coronary artery was seen in 3 patients (0.12 %). It originated from the right sinus of Valsalva in one patient and from LSV in two patients. In two other patients (0.08 %) the left coronary trunk originated from the RSV with separate ostium from the RCA. LCA originating from the pulmonary artery was found in one patient (0.04 %). A coronary artery fistula, which is a termination anomaly, was detected in 4 patients (0.15 %).

**Discussion:**

Although these anomalies, which are remarkably different from the normal structure, exist as early as birth, they are incidentally encountered during selective angiography or at autopsy. The incidence in reported angiographic series ranges from 0.6 % to 1.3 %. Variations in the frequency of primary congenital coronary anomalies may possibly have a genetic background. The largest angiographic series of 126595 patients, by Yamanaka and Hobbs, reported a 1.3 % incidence of anomalous coronary artery.

**Conclusion:**

The results of this study support the use MDCT coronary angiography as a safe and effective noninvasive imaging modality for defining CAAs in an appropriate clinical setting, providing detailed three-dimensional anatomic information that may be difficult to obtain with invasive angiography.

## Background

The anomalous origin of the coronary artery is a rare congenital condition with an incidence ranging from 0.17 % in autopsy cases [1] to 1.2 % in angiographically evaluated cases [2]. About 20 % of coronary anomalies produce life-threatening symptoms, including arrhythmias, syncope, myocardial infarction, or sudden death [3]. Coronary artery anomaly is the second most common cause of sudden cardiac death (SCD) in young athletes [4]. This imaging technique has limitations due to its projectional and invasive nature. The purpose of this study was to retrospectively determine the prevalence of origination, course, and termination anomalies of coronary arteries, CAA in subjects who presented different symptoms and underwent MDCT coronary angiography for the assessment of coronary artery disease.

## Methods

Between January 2008 and March 2012 a total number of 2572 consecutive patients were referred to the Euromedica-Kyanous Stavros Hospital, Department of Radiology, Thessaloniki, Greece, for cardiac MDCTA, due to suspicion(atypical stest pain, angina equivalent symptoms or multiple risk factors for cardiovascular disease) or assumed progression of coronary artery disease. Within these patients all datasets were reviewed in search of coronary anomalies of origin and further vessel course. Standardized patient preparation procedure included the administration of beta-blocker prior to the scan in order to stabilize and/or lower their heart rates below 65 beats per minute if needed (patients with HR > 70 bpm, and no contraindications). Additionally, patients sublingually received nitroglycerin immediately before contrast enhanced scan procedure to widen coronary arteries. An 18–20 gauge needle was placed into the antecubital vein, and heart rhythm was monitored by electrocardiography. MDCT coronary angiography was performed by using a 64-slice scanner (LightSpeed VCT 64 GE Healthcare device). The scan parameters were a collimation either 40 × 0.625 mm or 64 × 0.625 mm, rotation time 0.4 seconds, tube voltage 120 kV, and mAs 500–700. A bolus tracking technique was used to synchronise the arrival of contrast at the level of the coronary arteries at the beginning of acquisition. The images of the entire heart were acquired during apnea of 6 to 8 seconds, with intravenous infusion of 80 to 90 mL of iodinated contrast material, in infusion pump flow of 5 mL/s. A 30-mL bolus of normal saline was given after administering the contrast material to decrease the number of artifacts from the contrast material in the right heart. Two-dimensional maximum intensity projection, multiplanar reformatted and 3-D volume rendering images were produced throughout thin axial scans and then the coronary anatomy and arteries were evaluated. In all patients, images were reconstructed in end diastole (75 % of the R-R interval) and image quality was evaluated on a per segment basis. Where images were judged to be suboptimal, additional reconstruction windows (35 %, 65 % and 85 %) were explored in order to achieve optimal quality images. All analyses were performed on a dedicated workstation (Brilliance Workspace, GE Healthcare). All coronary MDCT coronary angiography images were assessed by an experienced radiologist who was blinded to the study and the whole data, figure and picture processing were approved by the Scientific Counsil of our hospital (reference number 13709).

## Results

Between January 2008 and March 2012 a total number of 2572 consecutive patients were referred for evaluation by MDCT coronary angiography. The patients were being tested for stent occlusion, for screening due to the presence of multiple risk factors, or due to chest pain, equivalent symptoms or inconclusive stress tests. Of the 2572 patients, sixty (2.33 %) were diagnosed with coronary artery anomalies, with a mean age of 53.6 ± 11.8 years (range 29–80 years), of which fifty (83.3 %) were males. The details of these coronary artery anomalies are summarized in the Table 1.

**Table 1:** Coronary artery anomalies encountered in our series

Type of anomaly	Number Of patients	Incidence (%)	Anomalies (%)
High “take-off”	20	0.78	33.3
Separate ostia for LAD and Cx inside the left SoV	15	0.58	25
RCA from LSV	9	0.35	15
Cx originating from the right SoV or RCA	6	0.23	10
Single coronary artery	3	0.12	5
LCA from RSoV	2	0.08	3.3
LCA from PA (ALCAPA)	1	0,04	1.7
Coronary artery fistulae	4	0.15	6.7
Total	60	2.33	

*LAD* left anterior descending, *Cx* circumflex, *RCA* right coronary artery, *LCA* left coronary artery, *SoV* sinus of Valsalva, *PA* pulmonary artery

**Fig. 1:**
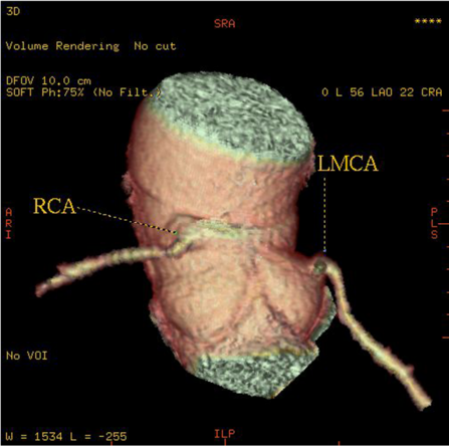
Volume-rendered image show high take-off of the right coronary artery (RCA) above the sinotubular junction

**Fig. 2:**
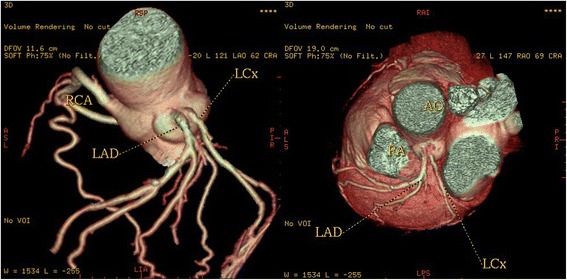
Volume-rendered images show the absence of the left main coronary artery, with separate ostia of the LAD and LCX arteries from the left sinus of Valsalva (LSV). A = aorta

**Fig. 3:**
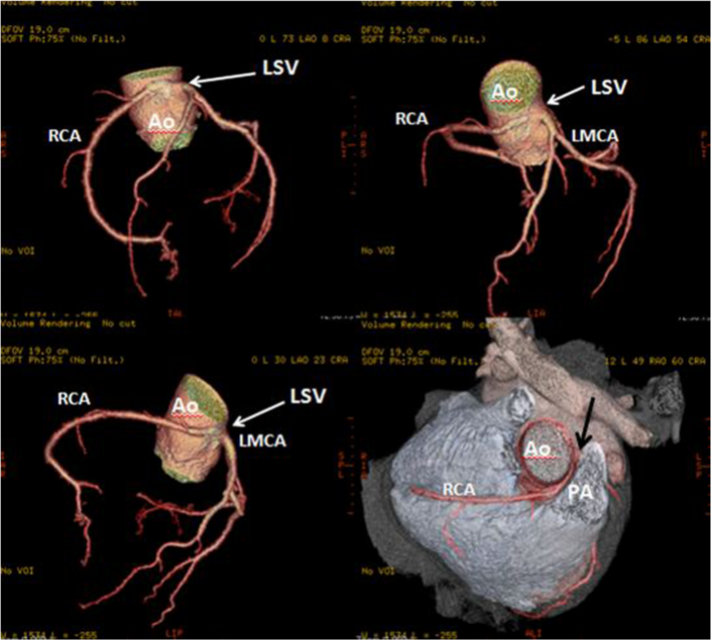
Three-dimensional volume-rendered image shows the RCA arising from the left coronary sinus and with a malignant inter-arterial course between the pulmonary artery (PA) and the aorta (Ao)

**Fig. 4:**
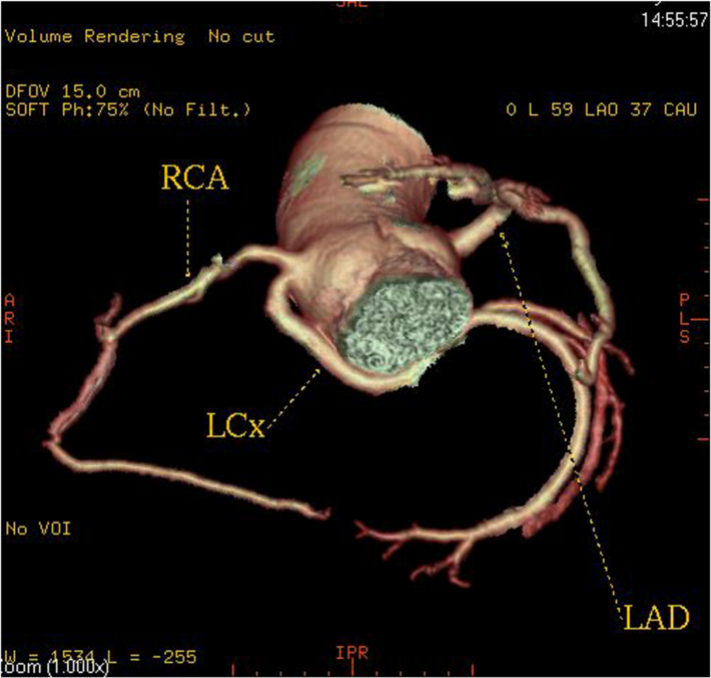
A patient with a left circumflex artery (LCX) anomalous origin. Three-dimensional volume-rendered image shows the LCX arising separately close to the origin of the right coronary artery (RCA) from the right coronary sinus and coursing below and behind the aortic root

High take-off of the right coronary artery (RCA) was seen in 16 patients (0.62 %), of the left main coronary artery (LMCA) in 2 patients (0.08 %) and both of them in 2 patients (0.08 %) (Fig 1). Separate origin of the left anterior descending artery (LAD) and left circumflex artery (LCx) from left sinus of Valsalva (LSV) was found in 15 patients (an incidence of 0.58 % or 25 % of all coronary anomalies) (Fig 2). In 9 patients (0.35 %) the right coronary artery (RCA) arose from the opposite sinus of Valsalva with a separate ostium for RCA and LM. In two patients the anomalous RCA had a malignant inter-arterial course (Fig 3). In 6 patients (0.23 % or 10 % of all anomalies) an abnormal origin of LCX from the right sinus of Valsalva (RSV) was found with a further posterior course within the atrioventricular groove (Fig 4). Termination of LCx was normal in all patients.

**Fig. 5:**
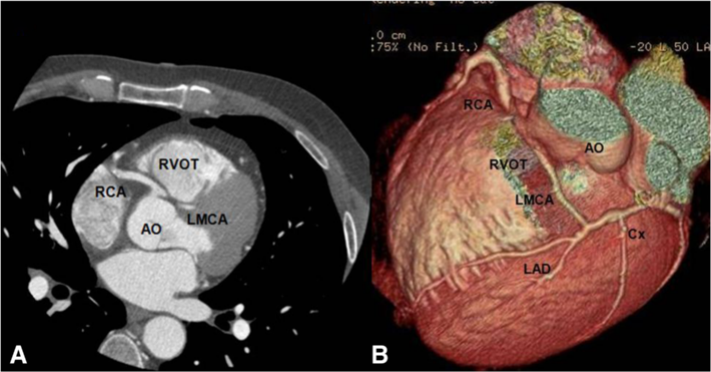
A patient with a single coronary artery originating from the right sinus of Valsalva. **a** Axial image demonstrating the left main coronary artery travelling between the aorta (AO) and the right ventricular outflow tract (**b**). Cardiac transparency image shows the anomalous origin of the LM, its course and the detailed anatomic relationship

**Fig. 6:**
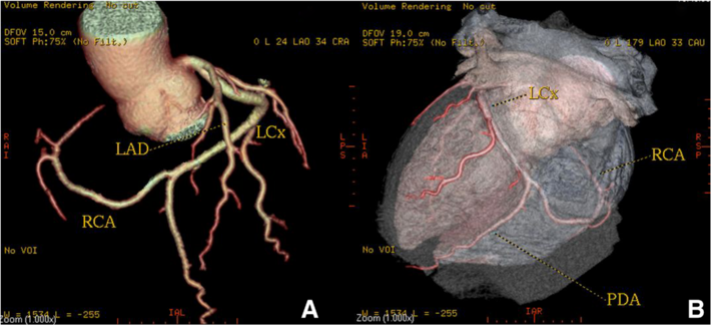
A patient with a single coronary artery. Volume-rendered and cardiac transparency images reveals a SCA arising from the left sinus of Valsalva and gives off the left anterior descending (LAD) and circumflex (LCx) branches. The right coronary artery ostium was congenitally absent (Panel **A**). The LCx is markedly dominant and continues beyond the crux into the atrioventricular groove and provides branches to the right ventricle and atrium (Panel **B**)

**Fig. 7:**
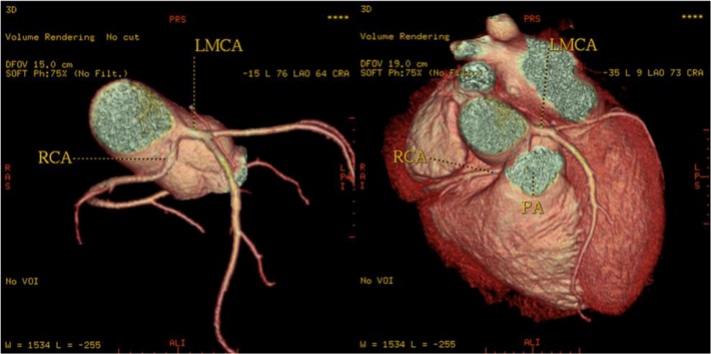
3-D volume-rendered images of the coronary tree showing a single coronary artery arising from the left coronary sinus, where the left main artery (LM) gives rise to the right coronary artery (RCA) as shown by the black arrow. The RCA runs a malignant course between the pulmonary artery (PA) and the aorta

A single coronary artery was seen in 3 patients (0.12 % or 5 % of anomalies). It originated from the RSV in one patient and from LSV in two patients. A 58-year-old man with positive exercise test was found to have a single coronary artery originating from the right sinus of Valsalva (Fig 5). The RCA gave rise to the LMCA, which had a course between the aorta and the right ventricular outflow tract (RVOT). In a 49-year-old man MDCTA revealed a single coronary artery arising from the left sinus of Valsalva (Fig 6). The right coronary artery ostium was congenitally absent. The LCx was markedly dominant and continued beyond the crux into the atrioventricular groove and provided branches to the right ventricle and atrium. In a 48- year-old man with history of syncope MDCT coronary angiography demonstrated a single coronary artery arising from the left coronary sinus, where the LMCA gave rise to the right coronary artery (RCA). The RCA had a malignant course between the pulmonary artery (PA) and the aorta (Fig 7).

**Fig. 8:**
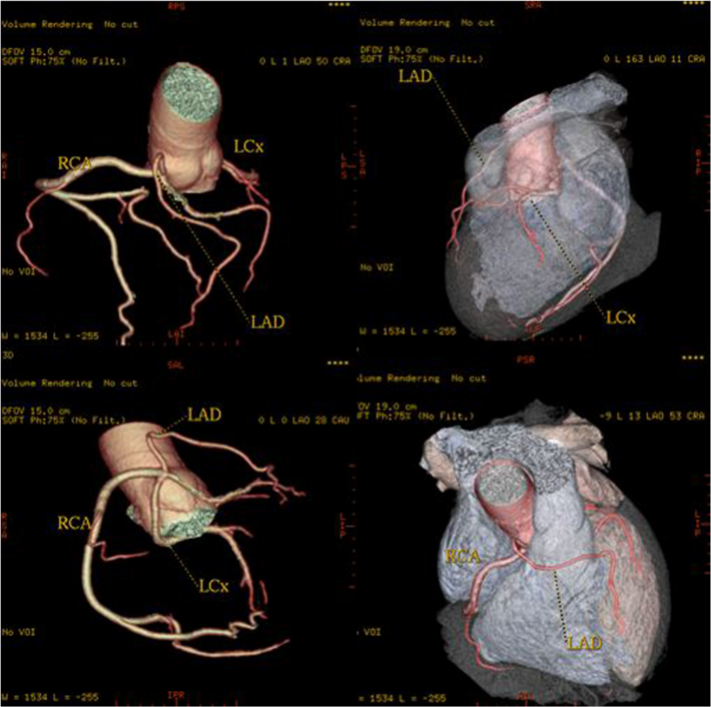
3-D Volume-rendered images, shows the entire coronary system originating from right sinus of Valsalva from three separate ostia. Left anterior descending coronary artery passes anterior to the right ventricular outflow tract. Circumflex artery passes posteriorly between the left atrium and the aortic root to resume its normal position in left atrioventricular groove. The Right coronary artery has normal configuration

**Fig. 9:**
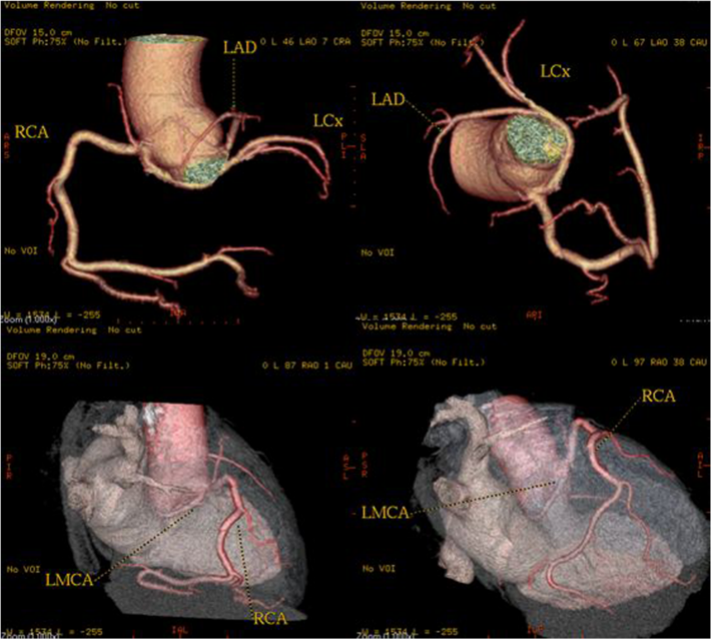
A patient with anomalous origin of the left coronary system from the right coronary cusp (separate ostium). 3-D volume-rendered and images show the anomalous origin of the LM, its course and the detailed anatomic relationship

**Fig. 10:**
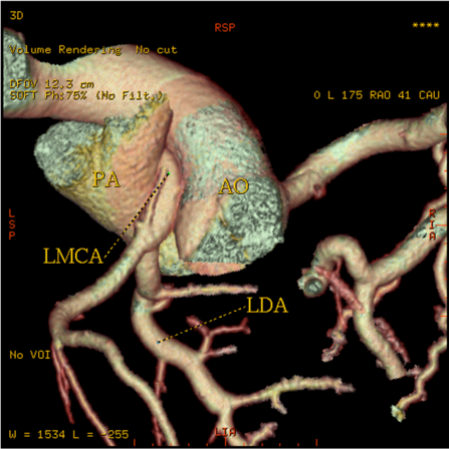
Three-dimensional volume-rendered image shows the dilated RCA from the aorta (Ao), the anomalous origin of the LMCA from pulmonary artery (PA), along with their anatomical relationship with surrounding structures. Rich collateral channels between RCA and LCA are shown

In 2 patients (0.08 % or 3.3 % of all anomalies) the left coronary trunk originated from the RSV with separate ostium from the RCA. In a 62-year-old woman the entire coronary system arose from right sinus of Valsalva from three separate ostia (Fig 8). The LAD passed anteriorly to the right ventricular outflow tract. The LCx passed posteriorly between left atrium and aortic root to resume its normal position in left atrioventricular groove. The RCA had normal anatomical apperance. A 49-year-old man with atypical chest pain was found to have anomalous origin of the left coronary system from the right coronary cusp (separate ostium), (Fig 9). LMCA from the pulmonary artery (ALCAPA) was seen in one patient (0.04 %). A 44-year old male with symptoms of heart failure was referred to our hospital for evaluation. MDCT coronary angiography demonstrated anomalous origin of the LMCA from the pulmonary artery and a dilated, tortuous and dominant RCA from the right aortic sinus, with profuse collateral channels feeding the left coronary system (Fig 10).

**Fig. 11:**
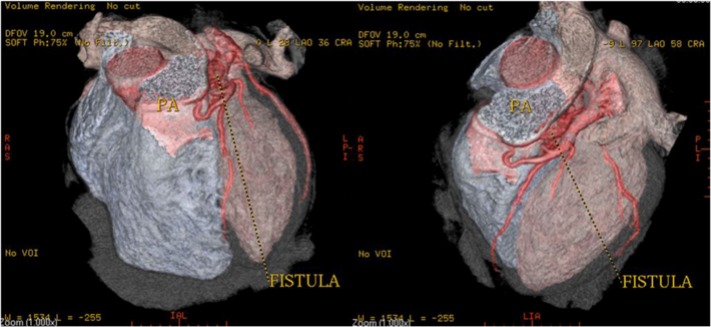
3-D volume rendering images showing the coronary fistula located between the left anterior descending (LAD) artery and the pulmonary artery

A coronary artery fistula, which is a termination anomaly, was detected in 4 patients (0.15 % or 6.7 % of all coronary anomalies). Three patients had coronary artery fistula between the left anterior descending artery and the pulmonary artery (Fig 11). In one patient the coronary artery fistula was located between the right coronary artery and the pulmonary artery.

## Discussion

Although these anomalies, which are remarkably different from the normal structure, exist as early as birth, they are incidentally encountered during selective angiography or at autopsy. The incidence in reported angiographic series ranges from 0.6 % to 1.3 %. Variations in the frequency of primary congenital coronary anomalies may possibly have a genetic background [5–10]. The largest angiographic series of 126595 patients, by Yamanaka and Hobbs, reported a 1.3 % incidence of anomalous coronary artery (8).

In our study, an overall of 60 patients (2.33 %) with CAAs were identified using MDCT coronary angiography. However there is a wide variety in the reported prevalence rate of CAAs [9–13]. De Jonge and co-workers also describe a prevalence of 7 % of CAAs including coronary fistulas [9] in their patient population. In one study the authors report a prevalence of coronary anomalies of origin and further course of 7.9 % in mainly symptomatic patients [10]. Very similarly, Shi et al*.* [11] demonstrated CTA to outperform invasive angiography for identifying the origin of anomalous coronary arteries and for confirming their anatomic course in relation to the great vessels. Of 240 patients, 16 (6.6 %) with anomalous coronary arteries were detected. In one study [12], anomalies were found in 44 (2.5 %) of 1,758 individuals undergoing MDCT coronary angiography. Recently, Srinivasan et al. [13] assessed 1495 patients using MDCT coronary angiography for the evaluation of coronary artery disease and found the prevalence to be 0.8 %. These discrepancies in reported prevalence might be caused by referral bias and lack of clear diagnostic criteria, which both are prerequisites for defining the true prevalence in a general population. Some of these patients with CAAs might have been or were referred because of known presence of CAAs and not because of unrelated factors as in the general population. In our study CAAs appear to be more common in men (*n* = 50; 83.3 %) than in women (*n* = 10; 16.7 %). This was also shown in previous reports although such a finding may reflect the selective nature of referral for cardiac MDCTA.

High take-off of coronary arteries was the most commonly seen coronary artery anomaly in this study (0.78 %). “High take-off” refers to an unusually high origin of either the RCA or the LMCA artery from the ascending aorta at a point above the junctional zone between its sinus and the tubular part [14]. The most common is a high origin of the RCA above the sinotubular junction. Rarely, the coronary artery can arise from the aortic arch, the brachiocephalic artery, the internal mammary, bronchial, or subclavian arteries, or even the descending aorta. High take-off positions are without any haemodynamic significance, well tolerated and asymptomatic, but they may lead to unexpected angiographic problems while localizing and engaging the orifices. High take-off is better represented on the angiographic view or volume-rendered reformatted images [15]. It is important for cardiac surgeons to be aware of this anomaly, because during cardiac bypass surgery when the aorta is cross-clamped, high cannulation is needed to avoid accidental cross-clamping or transection of the RCA.

The LAD and LCX arteries may arise separately from the left sinus of Valsalva (LSV) with an absence of the LMCA. (Estimated to be seen in 0.5 % to 8 % of population). This anomaly was the second most common anomaly in our series and was found in 15 patients (an incidence of 0.58 % or 25 % of all coronary anomalies). Multiple ostia [16] usually present no major clinical problems, but they may cause difficulty in cannulating the vessels during invasive coronary angiography.

With a prevalence ranging from 0.2 to 1.0 % [17], the anomalous origination of a coronary artery from the opposite sinus of Valsalva (ACAOS), represent a common cause of sudden cardiac death in the young population, particularly in individuals that undergo strenuous exercise [18]. In our series, similar to previous reports [6, 10], the prevalence of ACAOS was 0.78 % (33.3 % of all coronary anomalies), and 18 out of 20 patients were male. MDCTA allowed the identification of the ostium and proximal course of the anomalous coronary arteries and in addition, we were able to differentiate between high (i.e. interarterial) and low (i.e. retro-aortic) risk ACAOS. In our study a total of 6 patients with malignant inter-arterial course of the anomalous artery was found, either of the RCA (*n* = 4) or the left coronary artery (*n* = 2).

One of the most common coronary anomalies in our patient population was an anomalous RCA arising with separate ostium from the opposite sinus of Valsalva (*n* = 9, 0.35 %). Less often the RCA arises entirely from a single ostium in left coronary sinus (Fig. 7). The most common course of an anomalous RCA arising from the left sinus of Valsalva is interarterial [8]; this variant can be associated with sudden cardiac death in up to 30 % of patients [18]. In our series, four patients were found to have a malignant inter-arterial course of the anomalous RCA (Fig 3).

In this study, we report an incidence of 0.23 % for anomalous LCX, which account for a 10 % of the overall incidence of congenital coronary anomalies. The anomalous LCX artery always coursed posterior to the aorta to reach its normal distribution and its course was typical in all our patients. This anomaly alone causes no functional impairment of the myocardium, and it is therefore considered benign [19].

The single coronary artery refers to the origination of both the left and right coronary arteries from a single aortic ostium. In this study we observed three patients with single left coronary artery (0.12 % or 5 % of anomalies). It originated from the right Sinus of Valsalva in one patient and from LSV in two patients (in two patients, the anomalous artery had a malignant course between the pulmonary artery and the aorta).

Anomalous Origin of the Coronary Artery from the Pulmonary Artery is one of the most serious congenital coronary artery anomalies. In our study LCA originating from the pulmonary artery was seen in one patient (0.04 %). In rare instances the LCX or both the RCA and the LCA can originate from the pulmonary artery. Coronary artery origin from the pulmonary artery can occur as an isolated finding, though an associated cardiac abnormality, such as ASD, VSD, tetralogy of Fallot, aortic coarctation, double outlet right ventricle, and patent ductus arteriosus, can be seen in 5 % of cases [20, 21]. Extensive inter-coronary collaterals develop that are often dilated and tortuous. Symptoms usually occur due to coronary steal phenomenon caused by the flow of blood from the higher pressure coronary arterial system to the lower pressure pulmonary arteries. Surgical treatment is usually recommended for this anomalies [22].

A coronary artery fistula is an abnormal connection between one of the coronary arteries and another structure, most commonly a venous structure or a chamber on the right side of the heart. The prevalence is reported to be 0.002 % [23].

## Conclusions

The results of this study suggest that CT is a viable noninvasive modality for delineating coronary arterial anomalies. Knowledge of the CT appearances of various coronary artery anomalies and an understanding of the clinical significance of these anomalies are essential in making a correct diagnosis and planning patient treatment.
